# Multiparametric AFM reveals turgor-responsive net-like peptidoglycan architecture in live streptococci

**DOI:** 10.1038/ncomms8193

**Published:** 2015-05-28

**Authors:** Ron Saar Dover, Arkady Bitler, Eyal Shimoni, Patrick Trieu-Cuot, Yechiel Shai

**Affiliations:** 1 Department of Biological Chemistry, 8 Ulman Building, The Weizmann Institute of Science, Rehovot 76100, Israel; 2 Department of Chemical Research Support, Surface Analysis Unit, Goldwurm Building, The Weizmann Institute of Science, Rehovot 76100, Israel; 3 Department of Chemical Research Support, Electron Microscopy Unit, Issac Wolfson Building, The Weizmann Institute of Science, Rehovot 76100, Israel; 4 Department of Microbiology, Institut Pasteur, Unité de Biologie des Bactéries Pathogènes à Gram-Positif, CNRS ERL3526, Paris 75015, France

## Abstract

Cell-wall peptidoglycan (PG) of Gram-positive bacteria is a strong and elastic multi-layer designed to resist turgor pressure and determine the cell shape and growth. Despite its crucial role, its architecture remains largely unknown. Here using high-resolution multiparametric atomic force microscopy (AFM), we studied how the structure and elasticity of PG change when subjected to increasing turgor pressure in live Group B *Streptococcus*. We show a new net-like arrangement of PG, which stretches and stiffens following osmotic challenge. The same structure also exists in isogenic mutants lacking surface appendages. Cell aging does not alter the elasticity of the cell wall, yet destroys the net architecture and exposes single segmented strands with the same circumferential orientation as predicted for intact glycans. Together, we show a new functional PG architecture in live Gram-positive bacteria.

## Abstract (web summary)

The peptidoglycan (PG) layer of the Gram-positive bacteria cell wall resists turgor pressure, but the architecture of this layer is largely unknown. Here the authors use high resolution atomic force microscopy to image the PG layer from live *Streptococcus* to reveal a net-like arrangement that resists osmotic challenge by stretching and stiffening.

## Graphical abstract

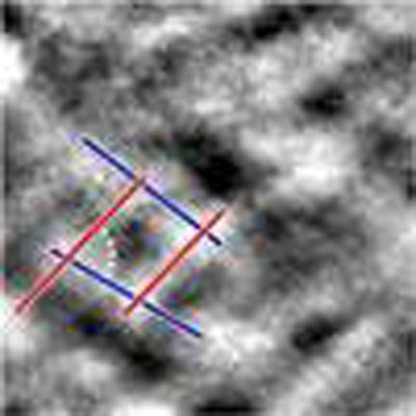

Gram-positive bacteria have a single cytoplasmic membrane, protected by a thick cell wall. Peptidoglycan (PG) is the major cell-wall structural element, as it forms a closed meshwork (sacculus) that provides a mechanical scaffold to support turgor pressure^1^,^2^. PG comprises of linear glycan strands, made up of N-acetylglucosaminyl-N-acetylmuraminyl disaccharides repeats that are cross-linked by short elastic peptide stems and together form a multilayered non-crystalline polymer^1^,^3^.

Glycans in elongated bacteria orient parallel to the membrane and around the cell's long axis (polar axis) to enable higher resistance to hoop tension^4^. Nevertheless, the nanoscale organization remains a subject of debate and different models have been proposed. For instance, an atomic force microscopy (AFM) study of purified sacculi from *Bacillus subtilis* found that PG forms 50-nm wide circumferential ridges, which are proposed to be helical cables formed by a few glycan strands^5^. A more recent electron cryo-tomography study of live bacteria, combined with computational simulation, found that the sacculus is uniformly dense and that glycan strands are actually aligned in parallel to each other^6^. A similar conflict is evident in ovoid bacteria as live-cell AFM revealed 25-nm wide PG bands that are thought to represent cables^7^, whereas PG of purified sacculi hardly form any surface structure^8^.

Here we studied live bacteria in different medium osmolarity, under an assumption that a change in turgor pressure should result in changes in the nanoscale surface architecture and elastic properties of PG. To study these changes simultaneously at high resolution, we used a multiparametric PeakForce tapping AFM (PFT-AFM, Bruker)^9^. The method enables higher sensitivity and minimal sample damage to soft biological samples due to a tight control over the force applied on the sample, as demonstrated in eukaryotic cells^10^,^11^, live bacteria^12^,^13^,^14^ and spores^15^, and recently reviewed by Dufrene *et al*.^16^. As a model organism for our investigation, we chose Group B *Streptococcus* (GBS, strain NEM316), an opportunistic pathogen that causes invasive infections in newborn children and either mortality or morbidity in adults with underlying diseases. Due to its clinical importance, the cell wall of GBS is well characterized and we used isogenic strains mutant in surface polysaccharides and proteins to ensure that structures are PG made. GBS lacks all members of the Mre cytoskeletal-like protein family^17^,^18^ and therefore the elastic properties of its cell wall are predominantly determined by the PG layer^19^,^20^.

We show that PG in live GBS forms a nanoscale net-like architecture made by parallel oriented glycans strands. The net stretches and stiffens to accommodate elevation in intra-cellular pressure.

## Results

### Morphological characterization of the cell wall

**Figure 1:**
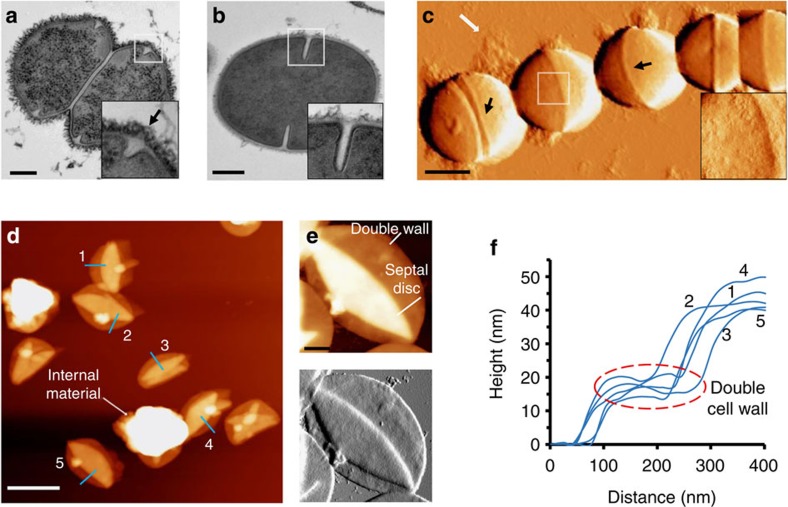
GBS cell-wall morphology. (**a**,**b**) Transmission electron microscopy images of **a** divided cell and **b** non-divided cell (scale bars, 200 nm). (**c**) Force-error AFM images of GBS chain in ambient conditions showing cell-wall molecules at the periphery (white arrow) and covering the surface (inset; scale bar, 500 nm). (**d**) Topography of isolated PG sacculi imaged by AFM in ambient conditions (scale bars, 1 μm; height, 85 nm). (**e**) Representative high-resolution topography (upper) and force-error (lower) images of sacculus do not show PG bands (scale bar, 150 nm; height of upper image is 50 nm). (**f**) Representative height profiles of PG sacculi corresponding to the blue lines in **d**. Black arrows in **a** (inset) and **c** mark the equatorial rings.

PG in GBS is covered by covalently bound pili proteins and two types of polysaccharides—10–45 nm long capsule (CPS) and group B carbohydrates (GBCs), which are branched molecules that function similar to wall teichoic acids of most Gram-positive bacteria^21^,^22^. Investigation of GBS using transmission electron microscopy demonstrate an ovoid (elliptic) morphology with poles that are round, but narrower than the cell diameter in both divided and single bacteria (Fig. 1a,b, respectively). The cell-wall surface is covered with fibrillar structures (Fig. 1a, inset), which were not evident in all cells (Fig. 1b, inset). These structures are characteristic of different surface proteins in *streptococci*^23^,^24^, which in our GBS serotype III may include the fibronectin-binding protein SfbA and R, C and X antigen proteins^24^,^25^,^26^. The GBC pellicle is heavily stained with metals and thus forms an outer dark layer^21^ (Fig. 1b, inset). The ridge-like feature named equatorial ring is denoted by an arrow and is an accumulation of new PG that marks the site of future division (Fig. 1a, inset)^8^,^21^,^27^. The PG layer is placed between the membrane (thin white line) and the GBC layer. Measurements taken from this region indicate an average of 31.9±6.3 nm (*n*=five measurements per cell, *N*=55 cells, ±s.d.). Our staining method does not show the capsule, but its presence is evident from AFM images of dry GBS chains at the cell periphery (Fig. 1c). The surface of the hemispheres is covered by a-periodic rough texture, which is likely a mixture of collapsed capsule molecules and pili (Fig. 1c, inset). Thus, PG cannot be imaged in dry bacteria.

To directly image PG, we scanned GBS sacculi that were isolated from polysaccharides and proteins (Fig. 1d). A total of 76 sacculi from 2 independent preparations were scanned and 53 of them were analysed using duplicated height measurements. Internal material that was not removed is often seen. The highest layer in clean sacculi is the collapsed septal disc (42.5±3.4 nm high). The double cell-wall height was on average 17.6±2.4 nm (Fig. 1f). We therefore account the thickness of a single cell wall as ∼9 nm. High-resolution scans of sacculi show a fairly homogenous surface, with no sign of periodic structures or bands that could hint on the PG arrangement (Fig. 1e). These findings are in good agreement to those reported in other ovococci by Wheeler *et al*.^8^, further supporting the idea that PG architecture is not comprised of large circular cables.

### PG in live GBS is stress stiffening

A recent study in live GBS using single-molecule force spectroscopy showed that PG is hindered by the capsule layer, when using low imaging force (0.25 nN), but not by the GBC layer or surface proteins^22^. Removal of the capsule revealed PG-associated surface architecture in the form of ∼25 nm circumferential bands proposed to be helical cables. However, we did not find these bands in isolated sacculi (Fig. 1f–h). A potential reason for this conflict may be that in isolated sacculi PG is relaxed and tightly packed^6^, while turgor pressure in live bacteria causes stress that stretches the material. Differences in PG density under mechanical stress were evident in sacculi of Gram-negative bacteria^28^. We therefore hypothesized that the PG architecture in live bacteria should expand under high pressure. Using multiparametric AFM enabled us to study whether structural alterations are coupled to changes in cell-wall elasticity.

To achieve high-resolution data, bacteria were immobilized in porous polycarbonate membranes and scanned using a 1 nN force. A fairly high force was necessary for two main reasons—first, it enabled us to penetrate the capsule and detect the PG bands on the surface of the wild-type (WT) strain. Second, accurate nanomechanical mapping using PFT-AFM requires surface indentation of at least a few nanometers^9^, as demonstrated in both Gram-negative^12^ and -positive bacteria^13^ and shown in Supplementary Fig. 1. We confirmed that such a force does not alter the surface topography during multiple scanning cycles (Supplementary Fig. 2).

**Figure 2:**
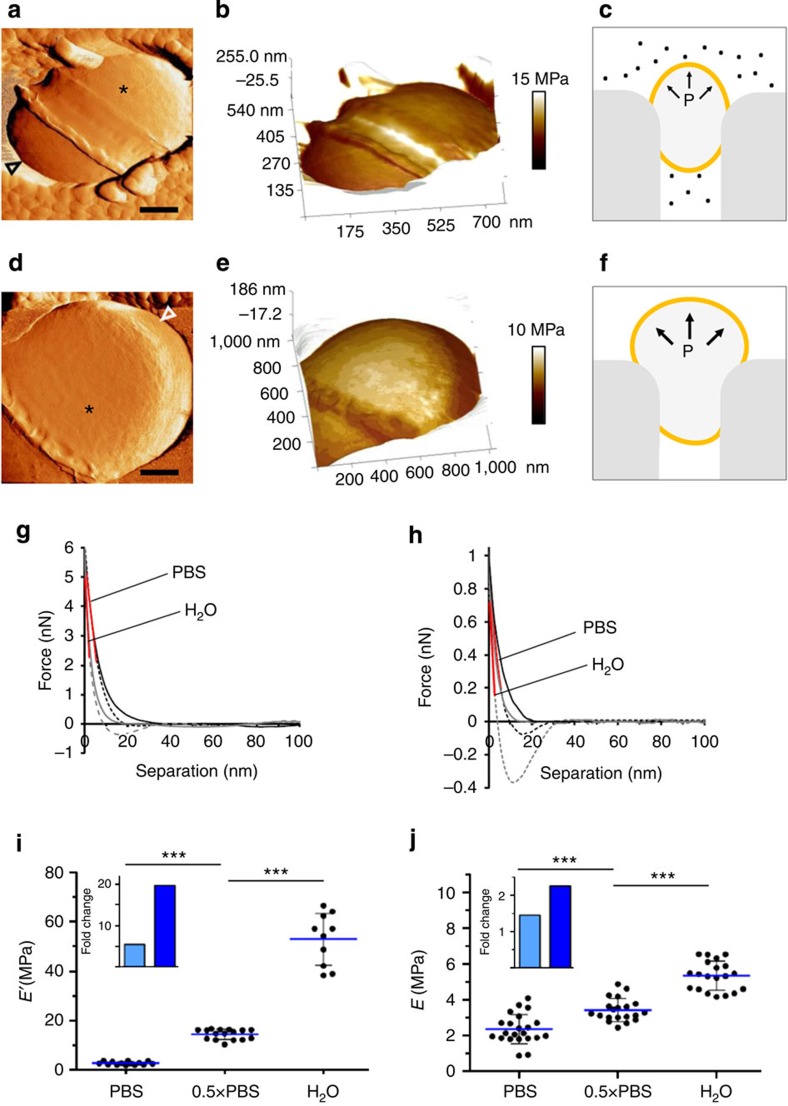
Elevation in turgor pressure reduces the cell-wall elasticity. (**a**,**d**) Force-error images of single pore-trapped GBS in PBS and in ultrapure water, respectively (scale bar, 200 nm). (**b**,**e**) Directly correlated elastic moduli calculated from a 1 nN peak-force scan and rendered on a 3D height representation of bacteria in PBS and ultrapure water, respectively. (**c**,**f**) Illustration showing pore-trapped bacteria in high or low medium osmolarity, respectively. Elevation in turgor pressure (P) results in a higher force that swells the exposed surface. (**g**,**h**) Representative force–distance curves recorded from a spot marked in the force-error images using **g** high and **h** low peak force (PBS represented by black line and water by grey line; Solid lines are approach and dashed lines are retraction curves; Red line is linear fit region, *R*^2^>0.98). (**i**,**j**) Elastic moduli of bacteria imaged using **i** 6 nN peak force (*E*′) and **j** 1 nN peak force (*E*) (average±s.d.; ***P value<0.0001, two-tailed unpaired *t*-test; insets show changes in elasticity in 0.5 × PBS (light blue) and water (dark blue) relative to PBS).

Changes in internal pressure were induced by changing the medium osmolarity^29^. Force-error images taken in physiological osmolarity (PBS) and in ultrapure water show single bacterium trapped along the polar axis (Fig. 2a,d, respectively). The influence of elevating the pressure is already assessed qualitatively from the fact that the exposed part of the cells was significantly larger in water than in PBS (Supplementary Fig. 3). A marked swelling in water is attributed to the fact that the cell volume is partially confined in a pore and only the exposed area is free to expand, similar to squeezing a balloon (Fig. 2c,f). It is intuitive that our system introduces a situation in which the exposed cell-wall surface may be overpressurized. Nevertheless, this serves our goal since local changes in PG structure are more likely to be detected.

Corresponding elasticity maps, rendered on a three-dimensional (3D) topography representation, demonstrate local changes in elasticity in the equatorial rings and the division site, while the surface of the hemispheres is fairly homogenous (Fig. 2b,e). Large differences in elasticity at the cell periphery is due to a sharp drop in height and therefore not included in our measurements^13^,^30^. Note that to better visualize small differences in surface elasticity, images were contrasted and the colour of the polycarbonate membrane clearly does not represent its true elasticity. To minimize the influence of surface curvature, measurements were taken from 150 nm^2^ areas at the cell's top, which correspond to at least 1,480 force–distance curves per bacterium. To ensure that our measurements reflect the elasticity of PG and not the resistance of the pressurized membrane to indentation, we scanned cells using low (1 nN) and high (6 nN) peak force, as recently done in *Staphylococcus aureus*^13^. Representative force–distance curves recorded on the hemispheres show a difference in the linear slopes (for example, spring constants) and deformation between PBS and water in both low and high indentation forces (Fig. 2g,h). These differences are translated to changes in the elastic modulus (*E*), which is the intrinsic stiffness of the material under a given stress^31^,^32^. Elastic moduli were calculated using the Derjaguin–Muller–Toporov (DMT) 33 fit to the linear part in the retraction curve. The DMT model assumes a spherical object indenting in the flat surface of an elastic half space. It is a safe approximation in our case since indentations are small, adhesion is weak and due to a difference of >2 orders of magnitude in curvatures of a bacterial surface compared with the AFM tip^13^. We keep in mind that absolute values are highly affected by the contact-mechanical model that is used, as well as by tip geometry and other imaging parameters. Therefore, given that identical imaging parameters are kept, data should be interpreted with respect to relative changes.

Bacteria from different cultures were imaged using high force (6 nN) or low force (1 nN) in three osmotic environments (PBS, 0.5 × PBS and water). Measurements using high force load show a drastic increase in elastic moduli when medium osmolarity is reduced linearly (*E*′_PBS_=2.68±0.63, *E*′_0.5 × PBS_=14.7±5.4 and *E*′_water_=52.9±10.3 MPa; at least 10 bacteria per condition; Fig. 2i), with a ∼20-fold difference between PBS and water (Fig. 2i, inset). Low-force measurements indicate a much more moderate, yet significant increase (*E*_PBS_=2.36±0.82, *E*′_0.5 × PBS_=0.42±0.65 and *E*_water_=5.33±0.81 MPa; at least 20 bacteria per condition) (Fig. 2j), with only a 2.25-fold difference between PBS and water (Fig. 2j, inset). Based on the findings that high force measurements result in much higher moduli in hypotonic medium, and on the fact that relative changes are much more pronounced compared with low-force measurements, we conclude that high force measurements are indication for elevated cellular pressure, while low-force measurements are sensitive to the intrinsic elasticity of PG. Interestingly, elasticity values obtained from these two measurements in physiological osmolarity are statistically identical (*P* value>0.05, two-tailed unpaired *t*-test), as recently reported in *S. aureus*^13^. This suggesting that only a little (undetectable) force is transmitted to PG by the membrane.

### PG forms a turgor-responsive net architecture

**Figure 3:**
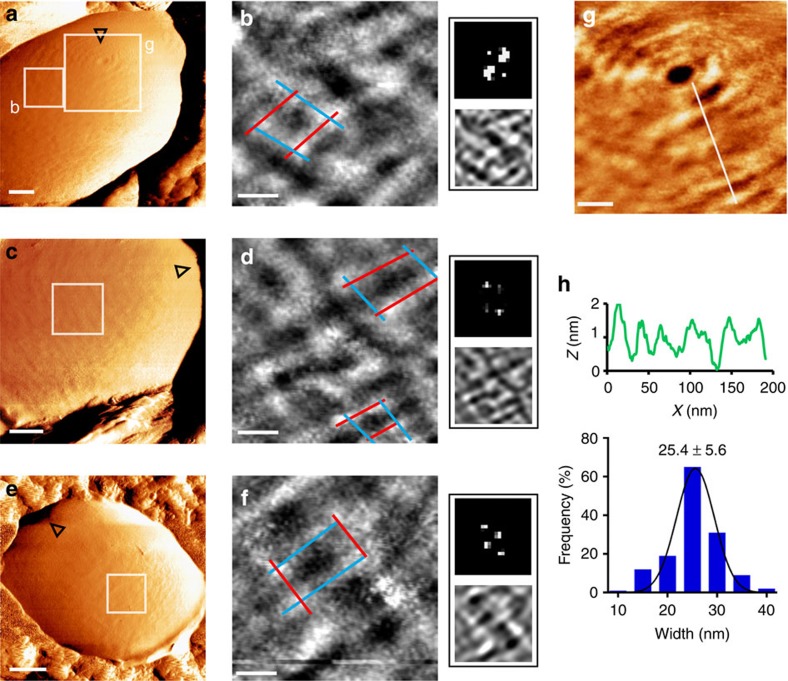
High-resolution imaging reveals a nanoscale net-like surface architecture. (**a**,**c**,**e**) Low-magnification force-error images recorded in PBS using 1 nN peak force show circumferential surface bands (scale bars, 100 nm; open triangles point towards the pole). (**b**,**d**,**f**) High-magnification height images show a net-like surface topography (scale bars, 30 nm; height, 1.2 nm). Colour lines demonstrate single holes formed by circumferential (blue) and connecting (red) bands. On the right of each image are a corresponding 2D-FFT map (upper) and a 2D-FFT filtered image (lower). (**g**) Height image, from a place marked in **a**, shows circular arrangement of bands around the pole (scale bar, 50 nm; height, 2.5 nm). (**h**) Representative height profile (upper) from a cross-section in **g**, and statistical analysis of band widths (histogram and an average±s.d.; *n*=35 bands per cell, from *N*=20 bacteria).

The changes in PG elasticity imply that in physiological osmolarity it is under low tensile stress, and less stretched than in hypotonic environment. To characterize the PG architecture in detail, we scanned the cell wall at very high resolution (∼1 nm per pixel), in PBS first. As mentioned earlier, we found that GBS display surface bands around the pole (Fig. 3a,g) or aligned in parallel to the division plane in the side wall (Fig. 3c,e). In agreement with the study by Beaussart *et al*.^22^, the average band width, as determined manually from height images, was normally distributed with an average of 25.4±5.6 nm (*n*=35 bands per cell from *N*=20 bacteria, Least squares Gaussian fit with *R*^2^=0.954; Fig. 3h). Surprisingly, a smaller-scale investigation revealed that bands are discontinuous and interconnected into a net-like structure that has not yet been described (Fig. 3b,d,f). Imaging different bacteria revealed that there is a certain degree of variability in the pore dimensions within each net and between nets of different bacteria, possibly due to differences in local surface tension and PG degradation. Nevertheless, all nets share common patterns—primary circumferential bands and connecting bands that are aligned roughly in a perpendicular orientation. To verify this notion, we isolated the dominant surface components from each image using 2D Fast Fourier Transformation (2D-FFT)^34^,^35^,^36^. The 2D-FFT algorithm detects periodic features by converting an image into a map of spatial frequency components^37^. Our maps show high-intensity ‘hot-spots' that form a twofold symmetry, corresponding to square or rectangular holes (Fig. 3b,d,f, upper right small images). 2D-FFT filtering removes very high frequency noise and shows the underlying structure of the net (Fig. 3b,d,f, lower right small images).

**Figure 4:**
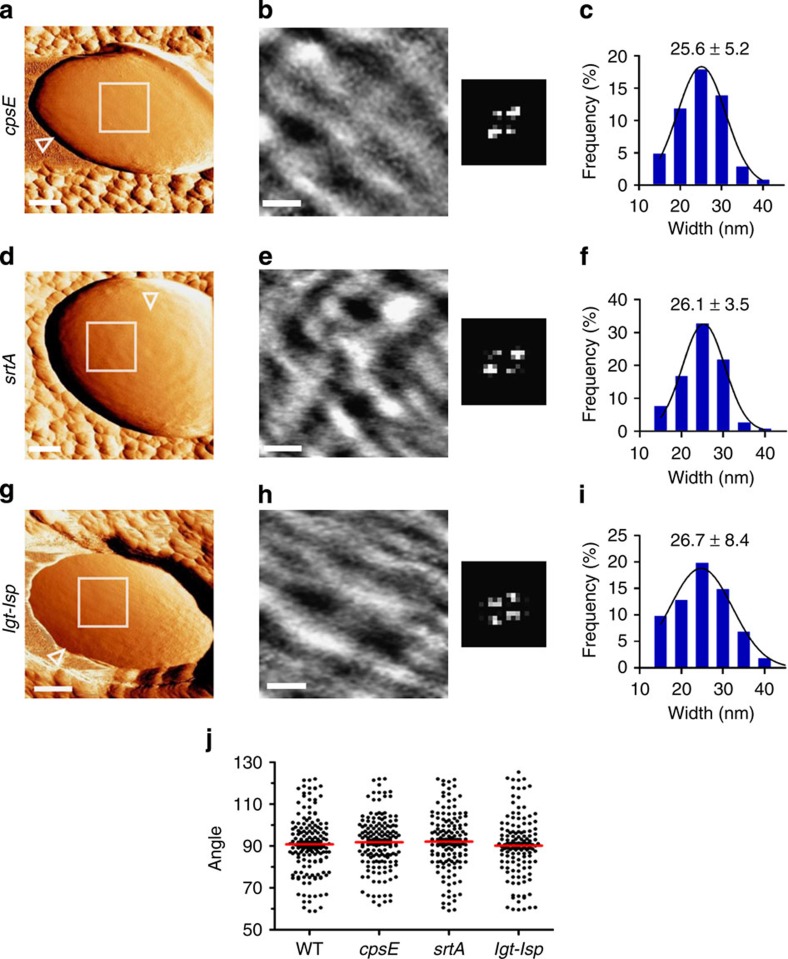
Mutants in surface molecules have similar surface structure as the WT strain. (**a**,**d**,**g**) Low-magnification force-error images recorded in PBS using 1 nN peak force from (**a**) capsule-deficient (*cpsE*), (**d**) cell-wall protein-deficient (*srtA*) and (**g**) membrane protein-deficient (*lgt-lsp*) mutants (scale bars, 100 nm; open triangles point towards the pole). (**b**,**e**,**h**) High-magnification height images show a net-like surface topography in every mutant strain (scale bars, 30 nm; height, 1.2 nm). Corresponding 2D-FFT maps are at the right side of each image. (**c**,**f**,**i**) Statistical analysis of band widths (histogram and an average±s.d.; *n*=35 bands per cell, from *N*=10 bacteria per strain). (**j**) A plot of all possible angles between circumferential and connecting elements of the net shows similar distribution in the WT and mutant strains.

To confirm that the net architecture is PG made and to determine whether surface appendeges may somehow affect the structure, we scanned isogenic mutants that are unable to: (i) synthesize the capsular polysaccharide (*cpsE*, formerly named *cpsD*), (ii) anchor proteins to PG (*srtA*) or (iii) synthesize membrane-bound lipoproteins (*lgt*-*lsp*)^21^,^38^,^39^. Similar to the WT strain, low-magnification force-error images show that all three mutants have a general banded surface (Fig. 4a,d,g), which forms a nanoscale net-like structure (Fig. 4b,e,h). To compare mutants to the WT strain, we quantitativly measured the width of the primary circumferential bands. In addition, for each image, we performed blind measurments of all the angles between the primary and connecting bands. At least 30 measurments per bacteria from at least 10 cells per strain were analysed and no significant difference between the WT strain and the mutants was found (*P* value»0.05, two-tailed unpaired *t*-test). Band widths distributed normally with an average of ∼25 nm (*cpsE*=25.6±5.2 nm, *srtA*=26.1±3.5 nm and *lgt-lsp*=26.7±8.4 nm, Least squares Gaussian fit with *R*^2^>0.955; Fig. 4c,f,i, respectively). Angle analysis revealed an avarage very close to 90° (WT=90.9±14.2°, *cpsE*=91.8±12.9°, *srtA*=92.1±14.3° and *lgt-lsp*=90.3±14.7°; Fig. 4j). However, as seen in the plot, variations from a straight angle are not rare and holes may have a diamond shape with acute and obtuse angles, as seen in the 2D-FFT images.

**Figure 5:**
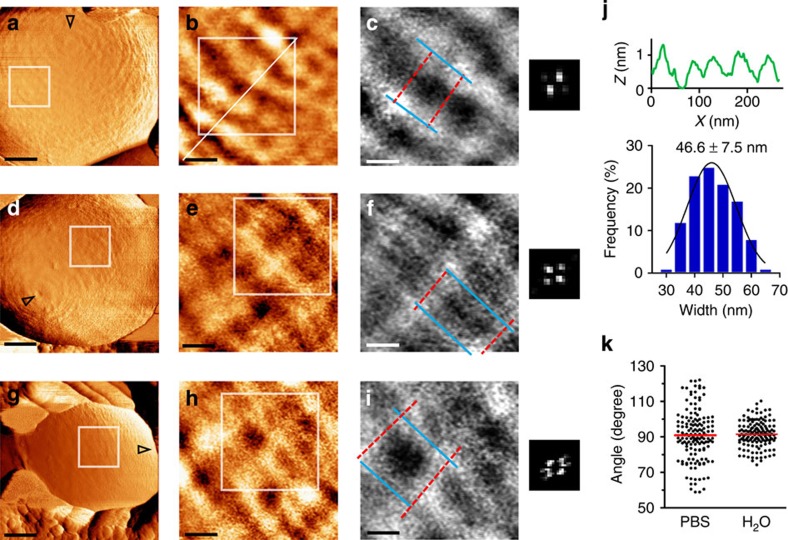
High turgor pressure stretches the peptidoglycan net. (**a**,**d**,**g**) Low-magnification force-error images recorded in ultrapure water (scale bar, 200 nm; open triangles point towards the pole). (**b**,**e**,**h**) Height images of surface structure (scale bar, 50 nm; height, 2 nm). (**c**,**f**,**i**) High-magnification height images show larger holes in the PG net (scale bars, 30 nm; height, 1.5 nm). Corresponding 2D-FFT maps are at the right side of each image. (**j**) Representative height profile (upper) from a cross-section in **b**, and statistical analysis of band widths (histogram and an average±s.d.; *n*=15 bands per cell, from *N*=18 bacteria). (**k**) Angles plot shows similar average but narrower distribution range in water compared with PBS.

The influence of pressure elevation on the PG net was significant. Bacteria imaged in water display circumferential bands that are already noticeable in low-magnification images (1 μm; Fig. 5a,d,g). A closer look at the surface topography shows a general net-like arrangement (Fig. 5b,e,h). However our measurements indicate that bands are about two times wider than those measured in PBS (46.6±7.5 nm, *n*=35 bands per cell form *N*=19 bacteria, Least squares Gaussian fit with *R*^2^=0.963; Fig. 5j). Measurements of hole angles in water indicate an average of 91.5±7.2°, which is statistically identical to that of bacteria in PBS (*P* value=0.642, two-tailed unpaired *t*-test; Fig. 5k). However, the s.d. is significantly lower (*P* value<0.0001) and therefore, angle distribution is more clustered around a straight angle, in agreement to the 2D-FFT maps.

Altogether, our data from live GBS reveal a new PG-made net-like surface architecture that stretches in response to elevated cellular pressure.

### Stationary bacteria display parallel twisted single strands

The net-like surface structure found in exponentially growing WT and mutants presumably represent intact cross-linked PG. To confirm that we investigated how cell-wall aging affects the surface structure by scanning bacteria from stationary cultures (OD_600_=1–1.2). A previous study in *S. aureus* showed that cleavage of the cross-linking pentaglycine bridges between glycan strands by lysostaphin significantly reduces the stiffness and causes major structural alterations to the cell wall^40^. A more recent study using PFT-AFM found that alterations in the secondary PG cross-linking significantly reduces the elastic modulus of the cell wall^13^.

**Figure 6:**
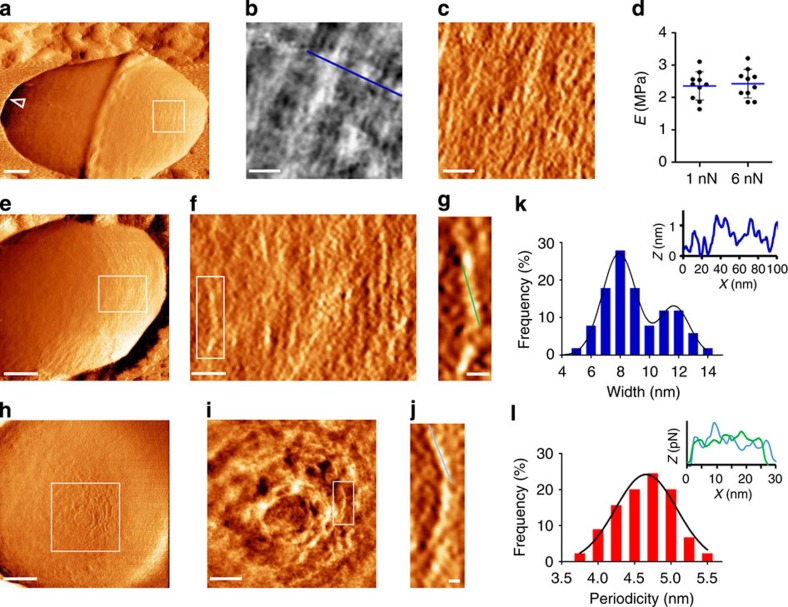
The surface architecture of stationary bacteria. (**a**,**e**,**h**) Low-magnification force-error images of (**a**,**e**) the side wall and (**h**) pole region (scale bars, 200 nm). (**b**,**i**) High-magnification topography images show nanoscale strands aligned in parallel or circling the pole (scale bars, 30 and 75 nm; heights, 1.4 and 2.35 nm, respectively). The strand widths were measured from height profiles (blue line in **b**,**k** inset), statistically analysed and is displayed in a histogram **k** (*n*=70 strands per cell, from *N*=8 cells). (**c**,**f**) Force-error images show that strands are segmented (scale bar, 30 nm). (**d**) Cell-wall elasticity, as measured using low and high peak force in PBS (*N*=10 bacteria from two different cultures, *P* value>0.05, two-tailed unpaired *t*-test). (**g**,**j**) Single strands from rectangles marked in **f**,**i**, respectively (scale bars, 10 nm). The segment periodicity was measured along each strand (corresponding profiles at the same colour are shown in the inset of **l**), statistically analysed and is displayed in a histogram **l** (*n*′=437 segments, *n*=55 strands from *N*=8 bacteria).

At the stationary stage, limitation in available nutrients promotes cleavage of the PG stem peptides by hydrolases to enable cell-wall turnover and possibly PG recycling^2^,^41^. Expectedly, elastic moduli calculated from stationary bacteria in PBS using low (1 nN) and high (6 nN) force were statistically identical (2.36±0.43 and 2.43±0.44 MPa, *N*=10 bacteria for each condition, *P* value>0.05, two-tailed unpaired *t*-test; Fig. 6d). However, this indicates that the cell wall of exponential and stationary bacteria has similar elastic properties, as was shown in *S. aureus*^30^. We assume that natural degradation, as opposed to exogenous addition of endopeptidase or mutation, is more moderate and perhaps compensated by stiffening of newer material or formation of alternative cross-linking^42^.

Despite the lack of detectable difference in elasticity, we found marked alteration in the surface topography in 8 out of 10 bacteria that were scanned at very high resolution (1,024 pixel per μm). These cells do not exhibit a banded structure, but rather have a rough surface (Fig. 6a,e,h). A high-magnification topographic view reveals strands that are arranged in parallel to the division plane (Fig. 6b) or around the pole (Fig. 6i). Similar surface structure was found in a stationary *srtA* mutant, indicating that these are not pili filaments or any other surface proteins (Supplementary Fig. 4). A Statistical analysis of data from topographic profiles (as demonstrated in Fig. 6b,k, inset) reveals two normally distributed sub-populations—a higher frequency population that has an average of 7.9±1.1 nm, and a lower frequency population that is on average 11.7±1.0-nm wide (*n*=70 strands per cell from *N*=8 cells, each strand was measured at two adjacent locations, data were fitted to a sum of two Gaussian distributions model; Fig. 6k). Force-error mode, which is more sensitive in lateral directions, shows that stands are segmented and adopt various twisted conformations (Fig. 6c,f). The strand periodicity, measured across the long axis of strands displaying distinct segmented structure, as demonstrated in Fig. 6g,j, is normally distributed with an average of 4.64±0.34 nm (*n*=55 strands in total, each strand was measured twice, least squares Gaussian fit with *R*^2^=0.972; Fig. 6h).

We propose that the observed strands are glycan chains that are expelled from the sacculus on degradation. Unfortunately, current high-resolution imaging techniques still lack the resolution to specifically identify small PG features in the context of a dense and cross-linked cell wall^22^. Therefore, our assertion is based on structural data and the fact that we found strands also in the stationary *srtA* mutant. Strands lay on the surface in an organized parallel orientation and are aligned in the same orientation as the PG bands of exponential bacteria. It is unlikely that capsule polysaccharides or pili proteins, which are filaments extending from the cell wall, would form such an organized surface structure. Previous AFM investigation of pili organization and assembly in *Lactobacillus rhamnosus* demonstrated that pili cannot be observed in liquid since they are too flexible or fragile^43^. Capsule molecules are also extremely flexible and form a smooth and featureless surface structure in liquid 22. When dried, cell-wall filaments collapse and create unordered structures on the cell surface, as we showed in Fig. 1c. We assume that the two sub-populations in our width analysis (Fig. 6k) represent glycans that are stretched to a different degree, due to different connectivity to the intact sacculus.

## Discussion

PG in Gram-positive bacteria is a multilayered polymer designed to resist the hydrostatic (turgor) pressure that pushes the membrane from within. Using multiparametric AFM investigation of live GBS, we showed a dynamic structural and mechanical response of PG to elevation in cellular pressure.

In agreement with previous findings in other ovococci^8^, purified sacculi of GBS are too thin and do not display banded structure as would be expected if PG was arranged as a large helical cable. High-resolution imaging revealed that the circumferential bands on the surface of live bacteria are in fact connected perpendicularly and form a net-like architecture that has not yet been reported. Imaging isogenic mutants confirmed that the dimensions of the elements of the net and its pore angles are not influenced by membrane proteins, cell-wall proteins or capsule and therefore we conclude that the net is PG made.

Under physiological salt concentration, the elastic modulus of the cell wall is similar to that of the whole-surface, as was recently shown in *S. aureus* by Loskill *et al*.^13^. This suggests that the force transmitted to the cell wall by the membrane is not detectable, and PG is in a less-stressed state. Reducing the medium osmolarity increases the internal pressure and consequently a higher stress is put on the PG layers. Respectively, the cell wall became less elastic and the PG net expanded as the width of the circumferential bands doubled and the pore angles became on average straighter in water. This is in agreement with Deng *et al*.^32^ who showed in *Escherichia coli* that both the cell radius and cell-wall stiffness are positively correlated to pressure and proposed a stress-stiffening response as a mechanism to limit shape changes under high pressure. A turgor-mediated increase in stiffness was also reported in yeast^44^.

Clearly, the net elements are too large to be single glycans and peptides. We propose that bands are formed by parallel glycan strands that are cross-linked by peptides into regions with higher and lower material density, which appear as bands and holes, respectively. Such a model better explains, in our opinion, the lack of distinct surface architecture in purified sacculi since loss of surface tension will reduce the differences between regions with different material density. An architecture of circumferential PG bands that contains more material seems to be a common feature of different Gram-negative species^28^, and despite the difference in chemical structure and thickness the essentials of our observation are similar. An nuclear magnetic resonance-based model of mixed close and open spaces was proposed in *S. aureus*^45^.

Further support to the model comes from aged cells. We found that the surface structure of stationary bacteria is not of a net, but rather of highly ordered parallel strands that are not cell-wall proteins and are aligned in parallel to the division plane or circle the pole. Strand periodicity is about 4.5 nm, only a little higher than that of fully relaxed helical glycans (4.1 nm), as calculated by X-ray scattering^46^, nuclear magnetic resonance^45^ and atomic-scale computer simulation^47^. The small size and high density of the strands currently prevent specific labelling, however, our structural data strongly suggest that these are glycan strands.

In summary, we show in live GBS a new nanoscale net-like arrangement of PG, which stretches and stiffens to accommodate elevation in cellular pressure following an osmotic challenge.

## Methods

### Bacterial culture and preparation

WT GBS (NEM316) and its isogenic mutants *cpsE*, *srtA* and *lgt- lsp* (see Supplementary Table 1) were grown in Todd Hewitt broth buffered with 100 mM HEPES to exponential (OD_600_=0.5) or stationary growth phase (OD_600_=1–1.4). Five millilitres of the culture were collected, centrifuged (3,000*g*, 5 min), and resuspended in 1 ml of 4 °C PBS (140 mM NaCl, 10 mM PO4 buffer, 3 mM KCl, pH 7.4). The procedure was repeated twice. The bacterial optical density was adjusted to OD_600_=1 (about 10^8^ cells per ml) at a total volume of 10 ml.

### Transmission electron microscopy

Exponentially growing cells were centrifuged (3,000*g*, 5 min) and the pellet was loaded on aluminium discs with depth of 100 mm (Engineering Office M. Wohlwend GmbH, Switzerland) and covered with a flat disc. The sandwiched sample was frozen in a HPM010 high-pressure freezing machine (Bal-Tec, Liechtenstein). Cells were subsequently freeze-substituted in a AFS2 freeze substitution device (Leica Microsystems, Austria) in anhydrous acetone containing 2% glutaraldehyde and 0.2% tannic acid osmium tetroxide for 3 days at −90 °C and then warmed up to −30 °C over 24 h. Samples were washed thrice with acetone, incubated for 1 h at room temperature with 2% osmium tetroxide, washed thrice with acetone and infiltrated for 5–7 days at room temperature in a series of increasing concentration of Epon in acetone. After polymerization at 60 °C, 60–80 nm sections were stained with uranyl acetate and lead citrate and examined in a Tecnai T12 electron microscope (FEI, Holland) operating at 120 kV, utilizing a 2k by 2k ES500W Erlangshen CCD camera (Gatan, UK).

### Preparing purified PG sacculi for AFM imaging

Bacteria were grown in 100 ml medium and collected in an exponential stage by centrifugation (3,000*g*, 5 min). Pellets were resuspended and boiled in 10 ml PBS for 7 min to avoid PG autolysis. About 20 ml PBS were added and the suspension was ice-cooled before bacteria were broken using French press (1,000 psi). Broken cells were collected (14,000*g*, 8 min, room temperature), resuspended in 5 ml of 6% SDS (pre-heated to 55 °C) and then boiled in water bath for 25 min. Insoluble material was collected by centrifugation (14,000*g*, 8 min, room temperature), resuspended in 5 ml of 4% SDS and boiled for another 15 min. The pellet was collected and SDS washed off six times with distilled water (60 °C).

To degrade proteins, pellets were treated with 2 mg ml^−1^ Pronase E (Sigma) in tris-HCl (50 mM, pH 7) at 60 °C for 90 min. Pronase E was inactivated by adding 4% SDS (final concentration) and boiling for 30 min. Insoluble material was collected and washed as described above. Polysaccharides were removed by overnight incubation with 48% hydrofluoric acid solution in 4 °C. Following acid treatment, pellets were washed once with Tris-HCl, and five times with distilled water until the pH reached neutral. Isolated sacculi were stored at −20 °C. For AFM experiments, sacculi were diluted in ultrapure water, deposited on freshly cleaved mica and dried overnight.

### Preparation of intact bacteria for AFM

Preparation of fixed-dried bacterial chains was as follows: a bacterial culture (OD_600_=0.5) was washed, resuspended in PBS and re-adjusted to OD_600_=0.5. Cells were immobilized for 20 min on a freshly cleaved MICA coated with poly-L-lysine (0.01 mg ml^−1^, Sigma), then washed to remove unattached bacteria and gently fixated with 1.5% glutaraldehyde for 10 min. Samples were washed again with double distilled water to remove glutaraldehyde traces and left to dry overnight at room temperature^38^. For imaging live bacteria in liquid, we separated GBS chains into single cells by two short cycles of sonication (Vibra Cell VCX 750; Sonics and Materials, Danbury, CT). Bacteria were sonicated at 30 W on ice for 10 s, with 30 s rest between intervals. We confirmed that cells remained undamaged using a SYTOX green staining for membrane perturbation^38^. A 10 ml suspension of single cells (OD_600_=1) was filtered through a porous polycarbonate membrane with a nominal pore size of 0.6 μm (Nucleapore, Whatman)^7^,^22^. Membranes were washed four times with ice-cold PBS to remove untrapped cells and attached to a metal disc using adhesive tab.

### AFM imaging

Images were acquired with MultiMode AFM (Bruker, Santa Barbara, CA) equipped with the Nanoscope V controller and a small scanner. For imaging dry sacculi, we used a 1 nN force, a scanning rate of 1 Hz and AC240TS cantilevers (Olympus) with spring constant of 2.6 N m^−1^. For imaging live bacteria, cells were imaged in PBS, 0.5 × PBS or MilliQ ultrapure water at room temperature (22–24 °C), in PeakForce QNM (quantitative nanomechanical mapping) mode^9^ using silicon nitride tip (Scan Asyst-fluid, Bruker). The spring constant of the cantilevers ranged between 0.55 and 0.68 N m^−1^, as determined by the thermal tune method for each experiment. The optical lever sensitivity was determined by pushing the cantilever against a hard surface (sapphire) before the experiments. The applied force was adjusted to 1 nN and the influence of the scan rate on image quality and mechanical parameters was assessed in different rates (1, 1.2, 1.4 and 2 Hz). No significant differences were found between scans and we used 1–1.2 Hz for further imaging at 1,024 pixels per μm resolution. The consistency and robustness of the structures was evaluated from scans at different scales and at perpendicular scanning direction. Numerical data presented is the mean value±s.d. Because our imaging medium does not prevent PG hydrolysis, membranes containing trapped cells were replaced after not more than 2 h of imaging^7^. Consequently, each data set is a collection of bacteria from different cultures obtained from different imaging sessions.

### Image and data analysis

Image processing and mechanical analysis was performed with the NanoScope Analysis software (Bruker). Small-scale height images were flattened using parabolic function to discard the curvature of the cell and reveal fine details. Quantitative data from the DMT modulus of elasticity mode was collected from 150 nm^2^ areas at the cell's top, from RAW images in duplicates. For 2D-FFT analysis, we used Gwyddion freeware. Statistical analysis was performed with GraphPad Prism 5, first using one-sided analysis of variance test and then by Student's unpaired non-parametric *t*-test for each pair of data sets. Significant difference was considered as *P* value<0.05.

## Additional information

**How to cite this article:** Saar Dover, R. *et al*. Multiparametric AFM reveals turgor-responsive net-like peptidoglycan architecture in live streptococci. *Nat. Commun.* 6:7193 doi: 10.1038/ncomms8193 (2015).

## Supplementary Material

Supplementary Information Supplementary Figures 1-4, Supplementary Table 1 and Supplementary References.

## References

[b1] Vollmer W. & Seligman S. J. Architecture of peptidoglycan: more data and more models. Trends Microbiol. 18, 59–66 (2010). 20060721 10.1016/j.tim.2009.12.004

[b2] Johnson J. W., Fisher J. F. & Mobashery S. Bacterial cell-wall recycling. Ann. NY Acad. Sci. 1277, 54–75 (2013). 23163477 10.1111/j.1749-6632.2012.06813.x PMC3556187

[b3] Cava F. & de Pedro M. A. Peptidoglycan plasticity in bacteria: emerging variability of the murein sacculus and their associated biological functions. Curr. Opin. Microbiol. 18C, 46–53 (2014). 24607990 10.1016/j.mib.2014.01.004

[b4] Turner R. D., Vollmer W. & Foster S. J. Different walls for rods and balls: The diversity of peptidoglycan. Mol. Microbiol. 91, 862–874 (2014). 24405365 10.1111/mmi.12513 PMC4015370

[b5] Hayhurst E. J., Kailas L., Hobbs J. K. & Foster S. J. Cell wall peptidoglycan architecture in *Bacillus subtilis*. Proc. Natl Acad. Sci. USA 105, 14603–14608 (2008). 18784364 10.1073/pnas.0804138105 PMC2567149

[b6] Beeby M., Gumbart J. C., Roux B. & Jensen G. J. Architecture and assembly of the Gram-positive cell wall. Mol. Microbiol. 88, 664–672 (2013). 23600697 10.1111/mmi.12203 PMC3663049

[b7] Andre G. . Imaging the nanoscale organization of peptidoglycan in living Lactococcus lactis cells. Nat. Commun. 1, 27 (2010). 20975688 10.1038/ncomms1027 PMC2964452

[b8] Wheeler R., Mesnage S., Boneca I. G., Hobbs J. K. & Foster S. J. Super-resolution microscopy reveals cell wall dynamics and peptidoglycan architecture in ovococcal bacteria. Mol. Microbiol. 82, 1096–1109 (2011). 22059678 10.1111/j.1365-2958.2011.07871.x

[b9] Pittenger, B., Erina, N. & Su, C. *Quantitative Mechanical Property Mapping at the Nanoscale with Peakforce QNM*. Application Note No. 28 (Bruker Nano Surfaces Divison, Santa Barbara, CA, USA, 2010).

[b10] Heu C., Berquand A., Elie-Caille C. & Nicod L. Glyphosate-induced stiffening of HaCaT keratinocytes, a Peak Force Tapping study on living cells. J. Struct. Biol. 178, 1–7 (2012). 22369932 10.1016/j.jsb.2012.02.007

[b11] Sharma S., Das K., Woo J. & Gimzewski J. K. Nanofilaments on glioblastoma exosomes revealed by peak force microscopy. J. R. Soc. Interface 11, 20131150 (2014). 24402921 10.1098/rsif.2013.1150 PMC3899882

[b12] Alsteens D., Trabelsi H., Soumillion P. & Dufrene Y. F. Multiparametric atomic force microscopy imaging of single bacteriophages extruding from living bacteria. Nat. Commun. 4, 2926 (2013). 24336094 10.1038/ncomms3926

[b13] Loskill P. . Reduction of the peptidoglycan crosslinking causes a decrease in stiffness of the staphylococcus aureus cell envelope. Biophys. J. 107, 1082–1089 (2014). 25185544 10.1016/j.bpj.2014.07.029 PMC4156677

[b14] Chen Y., Harapanahalli A. K., Busscher H. J., Norde W. & van der Mei H. C. Nanoscale cell wall deformation impacts long-range bacterial adhesion forces on surfaces. Appl. Environ. Microbiol. 80, 637–643 (2014). 24212582 10.1128/AEM.02745-13 PMC3911093

[b15] Pinzon-Arango P. A., Nagarajan R. & Camesano T. A. Effects of L-alanine and inosine germinants on the elasticity of Bacillus anthracis spores. Langmuir 26, 6535–6541 (2010). 20095533 10.1021/la904071y

[b16] Dufrene Y. F., Martinez-Martin D., Medalsy I., Alsteens D. & Muller D. J. Multiparametric imaging of biological systems by force-distance curve-based AFM. Nat. Methods 10, 847–854 (2013). 23985731 10.1038/nmeth.2602

[b17] Massidda O., Novakova L. & Vollmer W. From models to pathogens: how much have we learned about *Streptococcus pneumoniae* cell division? Environ. Microbiol. 15, 3133–3157 (2013). 23848140 10.1111/1462-2920.12189

[b18] Pinho M. G., Kjos M. & Veening J. W. How to get (a)round: mechanisms controlling growth and division of coccoid bacteria. Nat. Rev. Microbiol. 11, 601–614 (2013). 23949602 10.1038/nrmicro3088

[b19] Wang S., Arellano-Santoyo H., Combs P. A. & Shaevitz J. W. Actin-like cytoskeleton filaments contribute to cell mechanics in bacteria. Proc. Natl Acad. Sci. USA 107, 9182–9185 (2010). 20439764 10.1073/pnas.0911517107 PMC2889055

[b20] Lan G., Wolgemuth C. W. & Sun S. X. Z-ring force and cell shape during division in rod-like bacteria. Proc. Natl Acad. Sci. USA 104, 16110–16115 (2007). 17913889 10.1073/pnas.0702925104 PMC2042170

[b21] Caliot E. . Role of the Group B antigen of *Streptococcus agalactiae*: a peptidoglycan-anchored polysaccharide involved in cell wall biogenesis. PLoS Pathog. 8, e1002756 (2012). 22719253 10.1371/journal.ppat.1002756 PMC3375309

[b22] Beaussart A. . Molecular mapping of the cell wall polysaccharides of the human pathogen *Streptococcus agalactiae*. Nanoscale 6, 14820–14827 (2014). 25358409 10.1039/c4nr05280c

[b23] Swanson J., Hsu K. C. & Gotschlich E. C. Electron microscopic studies on streptococci. I. M antigen. J. Exp. Med. 130, 1063–1091 (1969). 5347694 10.1084/jem.130.5.1063 PMC2180487

[b24] Lindahl G., Stalhammar-Carlemalm M. & Areschoug T. Surface proteins of *Streptococcus agalactiae* and related proteins in other bacterial pathogens. Clin. Microbiol. Rev. 18, 102–127 (2005). 15653821 10.1128/CMR.18.1.102-127.2005 PMC544178

[b25] Flores A. E. & Ferrieri P. Molecular species of R-protein antigens produced by clinical isolates of group B streptococci. J. Clin. Microbiol. 27, 1050–1054 (1989). 2501340 10.1128/jcm.27.5.1050-1054.1989 PMC267481

[b26] Mu R. . Identification of a group B streptococcal fibronectin binding protein, SfbA, that contributes to invasion of brain endothelium and development of meningitis. Infect. Immun. 82, 2276–2286 (2014). 24643538 10.1128/IAI.01559-13 PMC4019170

[b27] Zapun A., Vernet T. & Pinho M. G. The different shapes of cocci. FEMS Microbiol. Rev. 32, 345–360 (2008). 18266741 10.1111/j.1574-6976.2007.00098.x

[b28] Turner R. D., Hurd A. F., Cadby A., Hobbs J. K. & Foster S. J. Cell wall elongation mode in Gram-negative bacteria is determined by peptidoglycan architecture. Nat. Commun. 4, 1496 (2013). 23422664 10.1038/ncomms2503 PMC3586723

[b29] Cayley D. S., Guttman H. J. & Record M. T. Jr Biophysical characterization of changes in amounts and activity of Escherichia coli cell and compartment water and turgor pressure in response to osmotic stress. Biophys. J. 78, 1748–1764 (2000). 10733957 10.1016/s0006-3495(00)76726-9 PMC1300771

[b30] Bailey R. G. . The interplay between cell wall mechanical properties and the cell cycle in *Staphylococcus aureus*. Biophys. J. 107, 2538–2545 (2014). 25468333 10.1016/j.bpj.2014.10.036 PMC4255174

[b31] Chang F. & Huang K. How and why cells grow as rods. BMC Biol. 12, 54 (2014). 25185019 10.1186/s12915-014-0054-8 PMC4243964

[b32] Deng Y., Sun M. & Shaevitz J. W. Direct measurement of cell wall stress stiffening and turgor pressure in live bacterial cells. Phys. Rev. Lett. 107, 158101 (2011). 22107320 10.1103/PhysRevLett.107.158101

[b33] Derjaguin B., Muller V. & Toporov Y. P. Effect of contact deformations on the adhesion of particles. J. Colloid Interface Sci. 53, 314–326 (1975).

[b34] Higgins A. M. & Jones R. A. Anisotropic spinodal dewetting as a route to self-assembly of patterned surfaces. Nature 404, 476–478 (2000). 10761910 10.1038/35006597

[b35] Coleman J. N. . Two-dimensional nanosheets produced by liquid exfoliation of layered materials. Science 331, 568–571 (2011). 21292974 10.1126/science.1194975

[b36] Baker A. A., Helbert W., Sugiyama J. & Miles M. J. New insight into cellulose structure by atomic force microscopy shows the i(alpha) crystal phase at near-atomic resolution. Biophys. J. 79, 1139–1145 (2000). 10920043 10.1016/S0006-3495(00)76367-3 PMC1301009

[b37] Watkins P. V., Kao J. P. & Kanold P. O. Spatial pattern of intra-laminar connectivity in supragranular mouse auditory cortex. Front. Neural Circuits 8, 15 (2014). 24653677 10.3389/fncir.2014.00015 PMC3949116

[b38] Saar-Dover R. . D-alanylation of lipoteichoic acids confers resistance to cationic peptides in group B streptococcus by increasing the cell wall density. PLoS Pathog. 8, e1002891 (2012). 22969424 10.1371/journal.ppat.1002891 PMC3435245

[b39] Konto-Ghiorghi Y. . Dual role for pilus in adherence to epithelial cells and biofilm formation in *Streptococcus agalactiae*. PLoS Pathog. 5, e1000422 (2009). 19424490 10.1371/journal.ppat.1000422 PMC2674936

[b40] Francius G., Domenech O., Mingeot-Leclercq M. P. & Dufrene Y. F. Direct observation of *Staphylococcus aureus* cell wall digestion by lysostaphin. J. Bacteriol. 190, 7904–7909 (2008). 18835985 10.1128/JB.01116-08 PMC2593208

[b41] Park J. T. & Uehara T. How bacteria consume their own exoskeletons (turnover and recycling of cell wall peptidoglycan). Microbiol. Mol. Biol. Rev. 72, 211–227 (2008). 18535144 10.1128/MMBR.00027-07 PMC2415748

[b42] Vollmer W., Blanot D. & de Pedro M. A. Peptidoglycan structure and architecture. FEMS Microbiol. Rev. 32, 149–167 (2008). 18194336 10.1111/j.1574-6976.2007.00094.x

[b43] Tripathi P. . Deciphering the nanometer-scale organization and assembly of *Lactobacillus rhamnosus* GG pili using atomic force microscopy. Langmuir 28, 2211–2216 (2012). 22149133 10.1021/la203834d

[b44] Arfsten J., Leupold S., Bradtmoller C., Kampen I. & Kwade A. Atomic force microscopy studies on the nanomechanical properties of Saccharomyces cerevisiae. Colloids Surf. B Biointerfaces 79, 284–290 (2010). 20452756 10.1016/j.colsurfb.2010.04.011

[b45] Kim S. J., Singh M., Preobrazhenskaya M. & Schaefer J. *Staphylococcus aureus* peptidoglycan stem packing by rotational-echo double resonance NMR spectroscopy. Biochemistry 52, 3651–3659 (2013). 23617832 10.1021/bi4005039 PMC3796188

[b46] Burge R. E., Fowler A. G. & Reaveley D. A. Structure of the peptidogylcan of bacterial cell walls. I. J. Mol. Biol. 117, 927–953 (1977). 606839 10.1016/s0022-2836(77)80006-5

[b47] Gumbart J. C., Beeby M., Jensen G. J. & Roux B. Escherichia coli peptidoglycan structure and mechanics as predicted by atomic-scale simulations. PLoS Comput. Biol. 10, e1003475 (2014). 24586129 10.1371/journal.pcbi.1003475 PMC3930494

