# Supplementary material for: Multiparametric AFM reveals turgor-responsive net-like peptidoglycan architecture in live streptococci
Source: Nat Commun. 2015 May 28;6:7193. doi: 10.1038/ncomms8193 (PMC4458890; doi:10.1038/ncomms8193)
Supplement: Supplementary Information — Supplementary Figures 1-4, Supplementary Table 1 and Supplementary References. [file ncomms8193-s1.pdf]

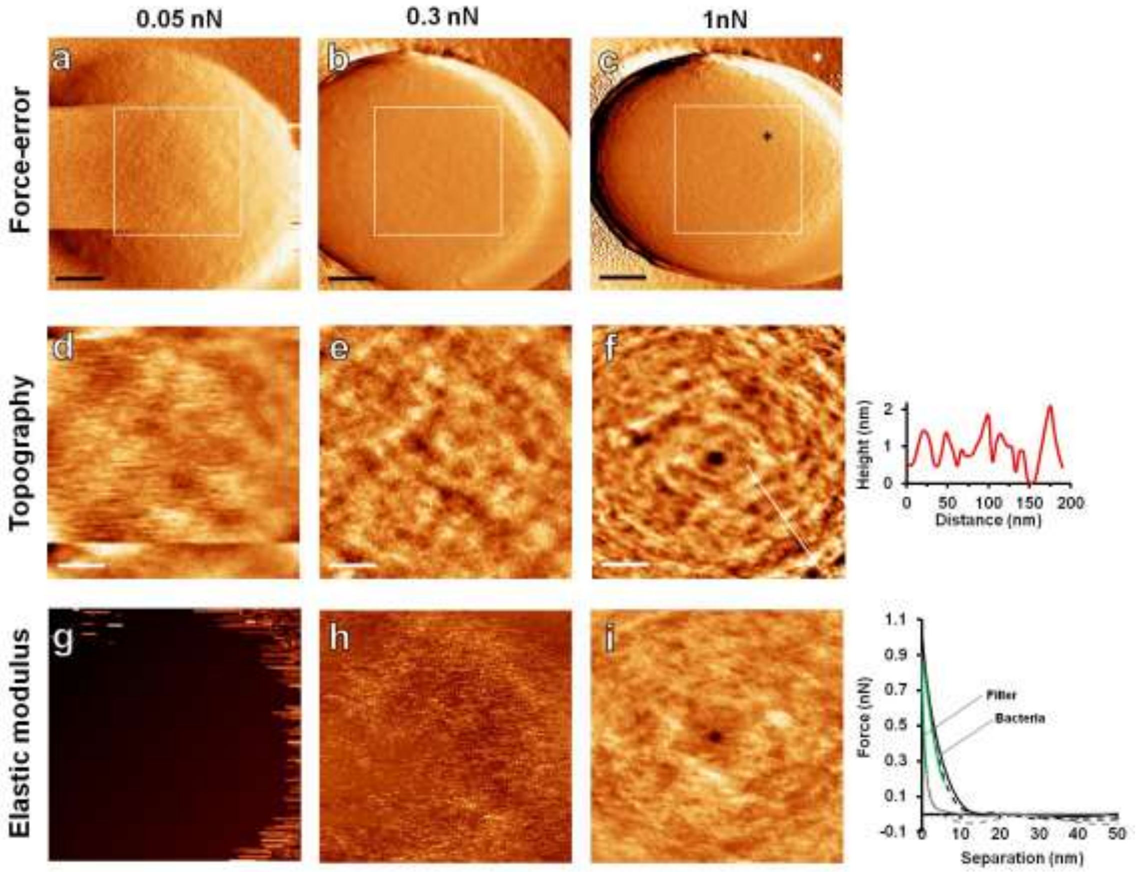

Supplementary Figure 1. The influence of different peak-force scans on surface architecture. (**a-c**) Peak-force error mode images of whole bacteria (scale bars, 200 nm). (**d-f**) Height mode images of the cell's top show a banded architecture only when a 1 nN imaging force is used (scale bars, 95 nm; height scale, 4.2 nm; profile of bands from **f** is at the right). (**g-i**) Elastic modulus images are sensitive to surface properties only when a 1 nN force is used (color scale, 0-4 MPa;). Force curves recorded from the cell (black) and filter (gray) from spots marked in **c** (approach is full line and retraction is dashed line).

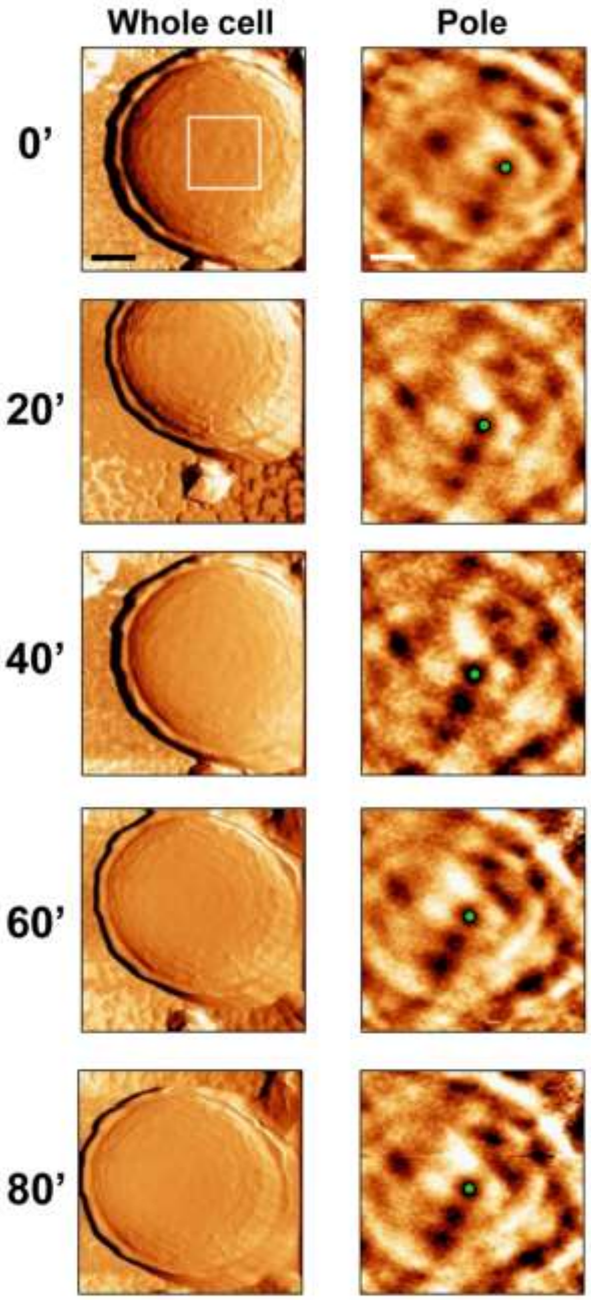

Supplementary Figure 2. Scanning bacteria using 1 nN force does not alter the surface topography during multiple scanning cycles. (a) Representative force-error images from an experiment in which single cells were scanned repeatedly in ultrapure water (scale bar, 200 nm; 1,024 pixle/µm; 1 Hz rate). (b) High-magnification height mode images show the surface topography does not significantly change with time (scale bar, 100 nm; height, 4.1 nm; green dot marks the center of the pole).

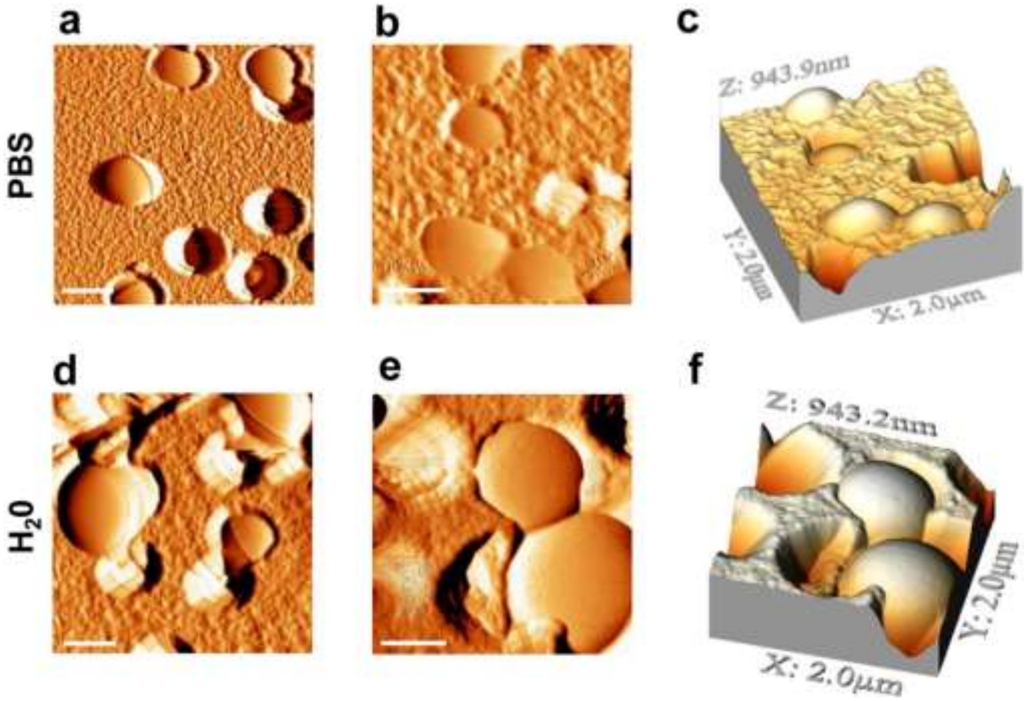

Supplementary Figure 3. Bacteria imaged in ultrapure water are significantly swollen. (**a**, **b**) Representative force-error image of pore-trapped GBS imaged PBS (scale bars, 600 nm and 500 nm, respectively). (**c**) 3D-representaion of **b**. (**d**, **e**) Representative force-error image in ultrapure water (scale bars, 600 nm and 500 nm, respectively). (**f**) 3D-representaion of **e**.

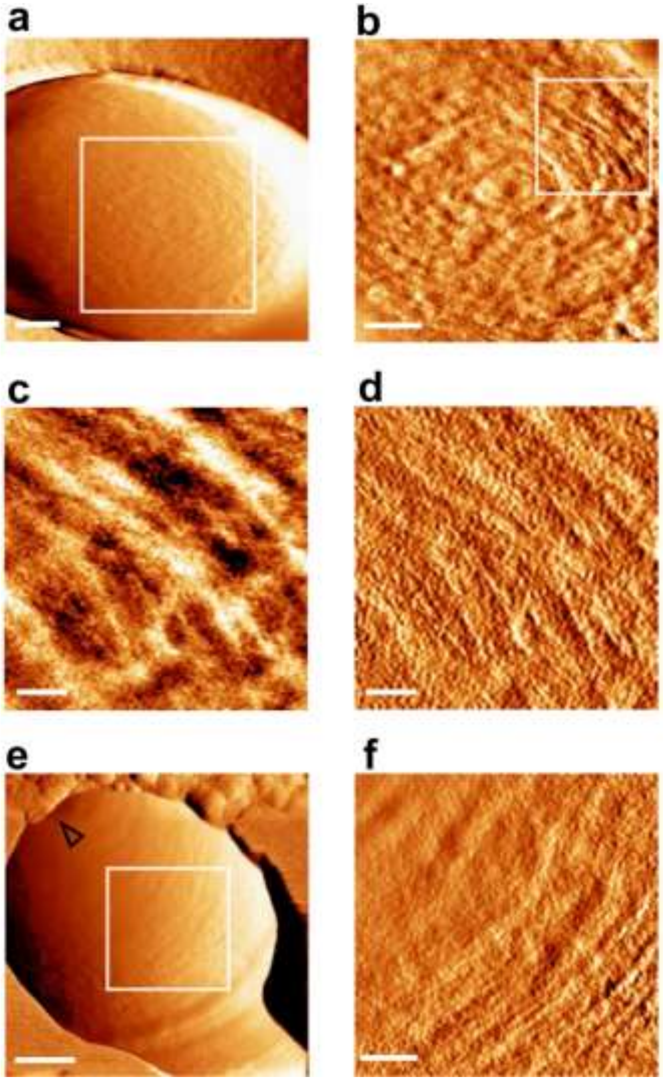

Supplementary Figure 4. Single strands in stationary *srt*A mutant. (**a, e**) Representative force-error image of stationary *srtA* mutant (scale bars, 150 nm and 200 nm, respectively). (**b**) Magnified force-error image of the pole region from **a** (scale bar, 75 nm). (**c, d**) Height and corresponding force-error images, respectively (scale bar, 25 nm; height, 1.3 nm). (**f**) Magnified force-error image of the side-wall region from **e** (scale bar, 25 nm).

Supplementary Table 1. Bacterial strains used in this study

Strains	Relevant genotype and characteristics[a]	Source
S. agalactiae		
NEM316	WT strain, serotype III, ST23	1
lgt-lsp	NEM316 *lgtΩaphA-3 lspΩaad6*; Km, Sm	2
cpsD	NEM316 *cpsDΩaphA-3*; Km	3
srtA	NEM316 *srtAΩaphA-3*; Km	4

[a]*aphA-3*, 3'-aminoglycoside phosphotransferase type III; *aad6*, 6-adenylyltransferase; Km and Sm, enzymatic resistance to kanamycin and streptomycin, respectively; Str, streptomycin-resistant mutant.

References

1. Glaser, P. et al. Genome sequence of Streptococcus agalactiae, a pathogen causing invasive neonatal disease. *Mol Microbiol* **45**, 1499-1513 (2002).
2. Henneke, P. et al. Lipoproteins are critical TLR2 activating toxins in group B streptococcal sepsis. *J Immunol* **180**, 6149-6158 (2008).
3. Poyart, C., Lamy, M.C., Boumaila, C., Fiedler, F. & Trieu-Cuot, P. Regulation of D-alanyl-lipoteichoic acid biosynthesis in Streptococcus agalactiae involves a novel two-component regulatory system. *J Bacteriol* **183**, 6324-6334 (2001).
4. Lalioui, L. et al. The SrtA Sortase of Streptococcus agalactiae is required for cell wall anchoring of proteins containing the LPXTG motif, for adhesion to epithelial cells, and for colonization of the mouse intestine. *Infect Immun* **73**, 3342-3350 (2005).
